# Clinical Pharmacy Intervention for Persons Experiencing Homelessness: Evaluation of Patient Perspectives in Service Design and Development

**DOI:** 10.3390/pharmacy7040153

**Published:** 2019-11-13

**Authors:** Parbir Jagpal, Nigel Barnes, Richard Lowrie, Amitava Banerjee, Vibhu Paudyal

**Affiliations:** 1 School of Pharmacy, College of Medical and Dental Sciences, University of Birmingham, Edgbaston, Birmingham B15 2TT, UK; P.K.Jagpal@bham.ac.uk; 2 Birmingham and Solihull Mental Health NHS Foundation Trust, Birmingham B23 6AL, UK; nigelbarnes@nhs.net; 3 NHS Greater Glasgow and Clyde, Glasgow, G76 7AT, UK; Richard.Lowrie@ggc.scot.nhs.uk; 4 Institute of Health Informatics, University College London, London NW1 2DA, UK; ami.banerjee@ucl.ac.uk

**Keywords:** homelessness, clinical pharmacy services, health inequality, public involvement and engagement

## Abstract

Persons experiencing homelessness have a high prevalence of severe mental health problems, alcohol dependence, substance misuse and infectious hepatitis C, and face up to twelve times higher mortality rates compared to the general population. They also face barriers to accessing healthcare. However, clinical pharmacy services are currently not available to homeless populations in England. The aim of this study was to conduct public involvement sessions with persons experiencing homelessness with a view to inform the design of patient-centred clinical pharmacy healthcare services. Qualitative methodology was used, using a focus group with homeless persons from emergency shelters and one to one engagement with those sleeping rough, using a topic guide. A total of nine homeless persons took part—seven males and two females. The participants of the sessions said that patient-centred clinical pharmacy services delivered for homeless persons would address many of their unmet needs around access to medicines, their understanding of prescribed medicines and holistic management of their health. The service would be able to make a positive impact on their health outcomes by screening for health conditions, facilitating better integration across services, referral and liaison with other services, and minimising misuse of prescribed medicines. The findings of this study will be used to inform the development, implementation and evaluation of a patient-centred clinical pharmacy service tailored to meet the specific needs of the homeless population.

## 1. Background

There are an estimated 250,000 people known to be homeless in England alone [1,2] including 4000 people sleeping rough on any given night [3]. The number of rough sleepers in urban areas such as London has doubled in the last 6 years [3].

People experiencing homelessness face up to twelve times higher mortality rates compared to the general population [4]. They die at an average age of 43 to 45 years [5]. A recent study conducted with over 900 homeless people in England showed a high prevalence of severe mental health problems, alcohol dependence, substance misuse and infectious hepatitis C [6]. A study in Scotland showed that the extent of multi-morbidity in homeless population, with a mean age of 40 years, was comparable to persons over 85 years in the general population [7]. 

Despite their poor health, people experiencing homelessness use health services differently to the general population [8]. Barriers include difficulty registering at a general practice due to lack of proof of address, inadequate signposting from healthcare staff, and perceived stigma and discrimination in healthcare settings [8,9]. They often lack the knowledge, and the physical and mental capacity to navigate services [8]. 

Homeless populations also experience reduced prescribing of medicines, and face barriers to accessing and managing their medicines. Cardiovascular diseases, mental health and substance misuse issues are often undiagnosed and under-treated [9,10,11]. Therefore, they have an increased rate of use of unscheduled care, with high resource and cost implications to healthcare services. There is a need for innovative service models to be tested and implemented, given the established link between homelessness and poor health. 

To facilitate engagement, fulfil the complex healthcare needs of homeless persons, and minimise access barriers, specialist primary healthcare centres for persons experiencing homelessness have been established across the UK. Such specialist centres aim to offer diverse services under one roof [12]. In Scotland, people who are homeless in Glasgow and Edinburgh have access to clinical pharmacists in the specialised homelessness health services and on outreach. However, clinical pharmacy services are currently not available to homeless populations in England, including those in the specialist settings. This is despite recent UK policies advising that the pharmacy workforce increase their role in reducing health inequality including amongst vulnerable groups [13,14]. Approximately 7500 (of the total 56,000 pharmacists in Great Britain) have qualified as an independent prescriber, allowing them to diagnose common medical conditions within their competency, prescribe medicines and make referrals. 

Recent work described the perceptions of people who are homeless of pharmacists delivering clinical services in Glasgow [15]. Findings included the service acting as a bridge into mainstream health services, and the pharmacists were reported to have improved patient health, while increasing patient perceptions of the importance of health [15]. 

This study aimed to conduct public involvement sessions using qualitative methodology to inform a proposal to develop a novel, patient-centred clinical pharmacy service for homeless persons in an English specialist homelessness health service and outreach setting, including temporary shelters and streets.

## 2. Method

A public involvement session using qualitative methodology was used. Patient and public involvement relates to input from members of public or patients in the actual design, undertaking, analysis or dissemination of findings, as opposed to their participation as research subjects. The National Institute of Health Research (NIHR) INVOLVE defines public involvement in research as activities being carried out ‘with’ or ‘by’ members of the public rather than ‘to’, ‘about’ or ‘for’ them [16]. Public involvement sessions are a pre-requisite as part of the submission of a research proposal, for funders including NIHR. 

This study was approved and funded by the National Institute of Health Research (NIHR) Research Design Service at University of Birmingham as a public involvement event, hence ethical approval was not required. However, informed written consent was obtained from focus group participants to record the conversation and use anonymised quotes for publication. Conversation was not recorded during public engagement with persons sleeping rough and only verbal consent was obtained.

A focus group and one to one engagement with persons currently experiencing homelessness were conducted. A focus group lasting approximately one hour was led by VP and PJ and was held at an emergency accommodation for persons experiencing homelessness. A total of ten registrants of a specialist homeless healthcare centre who had four or more prescribed medications were identified and invited by clinical staff at the specialist centre and were sent a letter of invitation to participate in the focus group. In addition, participants with multiple long-term health problems (as identified by shelter wardens—a list of conditions was not pre-specified) were also invited from the temporary shelter where the focus group took place.

The focus group was conducted in a communal area within the temporary shelter for people experiencing homelessness. Participants were provided with an information sheet in advance through postage or in person when invited to the event. The focus group was recorded and transcribed verbatim and analysed using thematic coding. 

Public engagement was also conducted with people sleeping rough. The researcher was accompanied by a charity worker and a substance misuse nurse for public involvement sessions in the street.

All participants were provided with a shopping voucher of £10 each, lunch, and reimbursed for any bus travel costs incurred. 

Both focus group and semi-structured interviews were conducted using a topic guide (Box 1) developed amongst the research team, based on the objective of the study and researchers’ previous experience around healthcare of homeless populations [8,9,17]. Data were analysed using thematic coding of the focus group transcript and notes taken during one to one engagement. Data saturation was not intended and detailed qualitative research methodology grounding was not considered in analysis, due to the adoption of public involvement methodology.

Box 1 **Study topic guide.**

- Why do you come to the specialist healthcare centre/use the services of the outreach team?
- Who would you expect to see/what types of staff—clinical/social care/support services?
- Have you faced problems in accessing health services before?
- Have you come across a pharmacist before?
- How do you think a pharmacist coming to you in the street as part of the outreach team or at the specialist healthcare centre can help you?
- Do you think having a pharmacist at the specialist healthcare centre /outreach team is a good idea?
- How do you feel about pharmacist doing health MOT for persons experiencing homelessness?
  E.g., screen for chronic health conditions each such as cardiovascular, respiratory, mental illness, anxiety and depression, alcohol and substance misuse, diet, podiatry, teeth
  - Medication review, adherence, starting/stopping medicines, monitoring
  - Signposting to other services [Prompt: how should they be doing it, where should they be doing it?]
- What issues do you face in getting and keeping the medicines you require?
- Do you often get enough time to discuss your medicines with your doctors, nurses?
- How do you feel about receiving support about your medicines from the pharmacist at the Health Exchange/outreach team? [prompt: optimising medicines, adding or reducing the number of medicines based on the checks, advice on storing medicines, getting your medicines, pharmacist liaising with the community pharmacist for you to get medicines on time, etc.]
- What differences (outcomes) do you think a pharmacist can make to a person experiencing homelessness?

## 3. Results

Altogether, nine participants took part in the public involvement events. This included seven (six males, one female) participants in the focus group, and two persons sleeping rough, including one female. The focus group and street engagement lasted approximately 1 hour and 20 minutes, respectively. Key themes from the discussion are presented below, with illustrative quotes presented from the focus group where available. Quotes are not available from individual engagement sessions with persons sleeping rough, as conversations were not recorded and consent to publish their direct quote was not sought, due to practicalities around health literacy and the consent process. 

### 3.1. Acquaintance with Pharmacy Services

Participants described using community pharmacy services for collecting their prescribed medicines. However, most of them described having a very low or no interaction with a community pharmacist when collecting their prescription. This lack of interaction was related to the busy environment of a community pharmacy. Minimal experience of pharmacist service in general practice was mentioned.

‘There wasn’t the opportunity to talk to the pharmacist … they don’t say, they just say a ten minute wait, that’s the only conversation you’re going to have.’

M 49

‘My experience is, it’s not good. You’re in a queue … And also, I put a prescription in on a Saturday, couldn’t get it till Monday so, I’d missed a couple of tablets anyway …’

M 56

### 3.2. Perceived Feasibility and Benefits of a Clinical Pharmacy Service 

Participants described their experience of visiting their doctor, nurse practitioners, substance misuse nurse and charity workers at the specialist healthcare centre, but not a pharmacist. Participants involved in the discussion mentioned that a clinical pharmacy service based at the specialist homeless healthcare would address many of the barriers homeless persons were facing around access to medicines and healthcare in general. 

a) Facilitating Access to Medicines

Participants described that they face a physical inability to visit a pharmacy, due to their multi-morbidities and illnesses and, hence, would often miss the collection of prescriptions. They would value clinical pharmacists’ help in facilitating timely collection of medicine at the specialist healthcare centre, as this was close to their temporary shelters.

‘I’ve got to walk on my heel sometimes, very painful, and if there was a pharmacist down there that’d save me a trip.’

M 50a

‘Yeah, I’ve missed medication cause I, I couldn’t get, cause I suffer with arthritis so, certain days I, it’s a no go even walking, I can’t walk…’

F 55

b) Understanding Prescribed Treatments 

Participants described that they often lacked understanding about their prescribed medicines. They would value somebody with expertise in medicine to ‘listen’ and develop a ‘rapport’ with them. One person described that often patients did not understand what was prescribed to them and there was not enough opportunity to query prescribing decisions by the doctors, due to lack of time. A clinical pharmacist based at the specialist primary healthcare centre would facilitate discussion of medicine-related issues. This would aid patient understanding of reasons for prescribing medication and its potential side-effects or interactions, and support better management of health conditions

‘I’ve recently started a new medication cause I was already diagnosed, dual diagnosis, when I was in prison I had an addition diagnosis, I started a new medication but, I’ve not had a chance to speak to anybody about the medication or potential side effects, whatever, whereas if there was a pharmacist there at the time, that would’ve helped a lot … and maybe if the doctor’s being awkward with you or you think that the doctors being unfair, if you’ve got a pharmacist to talk to, they can either back up what the doctor says or back up what you’re saying and then maybe they can go to the doctor. That would be good.’

M 50b

References were also made to the side effects of the medicines and potential role of pharmacists in preventing and managing the side effects.

‘When they put me on the medication in jail that was an anti-psychotic as well. So, I mean, and if you’re not the right person for that medication it can have really adverse effects. Luckily, I was the right person for it but…’

M 50b

c) Better Integration across Services

One participant also described a lack of liaison between primary and secondary care and prison services, which often lacked an effective transition of care, particularly with regards to prescribed medicines. A clinical pharmacist would be able to bridge such barriers.

‘I went to hospital once, cause I had an operation, it was stent, stent, yeah, got some but my doctor didn’t know about it until I look a letter to my doctor. He hadn’t had a copy so, he didn’t know what to prescribe me, he wasn’t even aware of the operation …‘. ‘I started heroin in jail cause they stopped my co-codamol. So, I pay for this, and my codeine.’

M 50a

d) Referral and Liaison with Other Services

Participants also mentioned that pharmacists would be ideal in facilitating their access and engagement with mental health services, as they had an expert understanding of medicines for mental health conditions and the negative impact of a disjointed approach towards the health and well-being of a patient. One of the persons sleeping rough mentioned that many homeless people were often reluctant to be admitted to hospitals, as many hospitals do not offer substance misuse services to inpatients. Clinical pharmacists would hence be effective in their referral process, by identifying and liaising with the ‘right’ hospitals and making sure that patients do not miss their prescribed treatments while in hospital.

e) Minimising Misuse of Prescribed Medicines

Participants described that misuse of prescribed medicines was often common amongst persons experiencing homelessness, as many of them had substance misuse problems. A pharmacist, as an expert in medicines who is based at the specialist centre, would be able to help address substance misuse by diagnoses, advice and referral to substance misuse services

‘… it helps them see if people are abusing their meds, know what I mean, they might see them taking too many straight away or selling on, stuff like that.’

M 50b

f) Screening, Diagnosis of Health Conditions and Prescribing of Medicines

Participants mentioned that they would trust pharmacists with screening for diseases, diagnostic skills and prescribing medicine and provided examples of their previous experience of pharmacists’ diagnostic skills.

‘Sometimes pharmacists are better at diagnosis than doctors, in my eyes.’

M (age not known)

‘I have one doctor saying I’ve got osteoarthritis, but my pharmacist said no, no, it’s rheumatoid, it is the other one, yeah … and the doctor said, no, it’s rheumatoid arthritis, you know, cause I worked in water, I was a plasterer by trade, so work in water over the years, hands shrank.’

M 50a

One rough sleeper mentioned that his blood pressure or any form of cardiovascular risk assessment was not done ‘for years’ as nobody had ‘come to’ him and that they were not registered with any general practice. One of the participants of the focus groups currently living in an emergency shelter also said that persons sleeping rough would most benefit from outreach visits.

‘Most people sleeping rough have got multiple problems going on but just don’t (have access to services). They seem to be the sort of people that don’t go to doctor’s appointments, don’t go and see doctors, don’t go and get it taken care of. Cause they’re living their day to day routine on the streets, whatever it is they have to do…they’re developing multiple physical or mental problems, but they (also) never search for any help so, a pharmacist going out to them, they’d be getting the care and attention that they should be getting but, they’re choosing not to for whatever reason. They probably get, they’d probably benefit more from it than anyone.’

M 50b

### 3.3. Prospect of Outreach Visits by Pharmacists

Participants suggested that pharmacists could visit persons experiencing homeless in temporary shelters, emergency accommodation and streets during outreach. They mentioned that some patients may be unable to walk to the specialist primary healthcare centre or make appointments and hence outreach would ensure continuity of care.

‘They (persons experiencing homelessness) don’t go to a doctor … so, if you went out to them … they would probably open up to you.’

F 55

### 3.4. Peer Support and Social Influences in Engaging with Services

Participants mentioned that social influences were key in encouraging people sleeping rough to engage with outreach services by pharmacists.

‘And when one person hears from another that they, they sent the wellbeing bus or whatever and it helps them, and they move on or they got better. Word of mouth alone is going to build trust amongst these people.’

M 50b

One participant suggested that a peer support network would facilitate engagement with the outreach services.

‘Suggesting recruiting people like us, who are already on the street, some of us, going to know certain people, and I’m just getting nowhere, here mate, I’ll signpost you to them. I mean what is it really, are you going to go out yourself or you’re going…’

M 50a

### 3.5. Addressing Challenges in Following up Homeless Persons

Participants were requested to suggest ways to address the challenges to follow up people experiencing homelessness during their care. They mentioned that building trust and rapport means participants were more likely to agree a mutual meeting point either at the specialist homeless healthcare centre, at the temporary shelter or at a natural venue. 

## 4. Discussion

Public involvement sessions were held with persons experiencing homelessness with a view to inform the development, implementation and evaluation of patient-centred clinical pharmacy interventions for homeless persons. The participants of the session described that a clinical pharmacy service tailored to this specialist population was a feasible idea that could deliver benefits to the patients. However, the low sample size of this study means that study results are not generalizable and there is a need to develop, implement and evaluate the intervention to identify the evidence base. This would address the unmet needs of homeless persons around access to medicine, support their understanding of prescribed medicines and improve health outcomes. Participants suggested that the best way to deliver primary healthcare to people sleeping rough was to promote engagement by building trust, and for the health service to ‘reach out to them’ when needed.

Participants of the sessions trusted pharmacists to screen for health and long-term conditions and provided examples to back up their assertions. A novel, pharmacist-led health outreach service for homeless people has been delivered and evaluated in Glasgow [18]. In this study, pharmacists provided pop-up, drop-in (no appointment needed) health clinics at various homeless support venues. Medications were prescribed by pharmacists in 62% of all patients, of which over two thirds were new medications. New clinical issues were identified in 69% of patients. More importantly, this exploratory study showed an improvement in patient engagement with the service, with 85% of patients subsequently attending either a follow-up with the pharmacist or with other referred services. It is proposed that the service for England should similarly be able to introduce screening for cardiovascular health, blood borne viruses, and sexual health.

There is very little published literature on clinical pharmacy services for homeless persons from outside the UK. A study conducted in the US [19], which included pharmacists and pharmacy students offering free primary healthcare services to 100 homeless persons, showed that the pharmacy team were able to diagnose new health conditions including diabetes, hypertension, and hyperlipidaemia, and identify medication-related problems, which mostly included under-prescribing or ineffective drug prescribing. The pharmacy team also facilitated access to free medicines in a private healthcare system. Data collected six months prior to and after the implementation of the services showed an improvement in clinical outcomes for patients enrolled in the clinical pharmacy programme. Statistical comparisons were not available, due to limited sample size. Another observational study in the US also demonstrated pharmacists’ contribution to reducing medication-related issues in homeless persons, which mostly related to under-prescribing, inappropriate medication prescribing and suboptimal doses [20].

A systematic review of the international literature, which did not include the aforementioned studies, demonstrated a dearth of, and, hence, a need to generate, evidence for the management of communicable and non-communicable diseases in persons experiencing homelessness [21]. The review showed that, of the 11 included studies, none of the previous studies included clinical pharmacy interventions.

Some of the outcomes of the public involvement sessions corroborate with our recent qualitative study that aimed to investigate the barriers for people experiencing homelessness to access health services [8]. They experienced difficulty registering at a general practice due to lack of proof of address, inadequate signposting from healthcare staff, and perceived stigma and discrimination in healthcare settings. They often lacked the knowledge, and the physical and mental capacity to navigate services, which were often deemed to be fragmented. These views were also shared by participants in the public involvement sessions who were currently homeless. Wider policy and practice changes are needed to promote service integration [22]. 

Participants in these public involvement sessions said that they would trust pharmacists to prescribe medicines to them. Cardiovascular diseases, mental health and substance misuse issues are often undiagnosed and under-treated [6,10,11], and pharmacists have the potential to address these issues.

### 4.1. Strengths and Limitations

To our knowledge, this is the first report of a public involvement session that included persons experiencing homelessness to discuss their suggestions on the value and nature of clinical pharmacy services. Engagement can be difficult, as persons experiencing homelessness lack stable accommodation. Meeting participants in emergency accommodation, or on the street with healthcare workers that they are already familiar with, provides a safe environment in which to explore their views.

Given that the study was focused on an under-researched area, an exploratory approach using qualitative methodology was considered most appropriate, as these are suited over survey designs which mainly serve ‘confirmatory’ purposes. Homeless persons are often a hard to reach population and it is a norm for a small sample size to be utilised in research involving homeless persons. We did not subject a recruitment strategy for data saturation. Therefore, the findings of this study are not meant to be generalizable to the wider homeless population in the UK.

During the discussion, the researchers introduced themselves as being affiliated to the pharmacy discipline and, therefore, there may have been some degree of social desirability bias [23] in participants’ responses to the questions. However, to minimise such bias, the researchers assured participants that there were no right or wrong answers to any questions prior to the start of the interviews, with researchers interested in a range of views on the topic of discussion. 

### 4.2. Future Plans

Suggestions for clinical pharmacy interventions are being incorporated into intervention design and development. Funding will be sought from relevant agencies in the development, implementation and evaluation of services. Educational interventions to support pharmacists in other settings, such as community pharmacy, is also required to promote joined-up working [24]. 

## 5. Conclusions

Public involvement sessions were conducted to inform the feasibility, design and development of clinical pharmacy intervention for persons experiencing homelessness. The outcomes provide recommendations that persons experiencing homelessness perceive that a clinical pharmacy service would bring multiple benefits around access to and use of medicines, and facilitate access and integration of services for person-centred care. The findings will be used to develop a feasibility and pilot intervention that aims to explore the benefits of clinical pharmacy service in primary care to the health of persons experiencing homelessness. 
